# Case Report: Abnormally low hemoglobin A1c in a diabetic patient with *SLC4A1* gene mutation

**DOI:** 10.3389/fendo.2026.1785435

**Published:** 2026-04-14

**Authors:** Lili Ye, Qian Ren, Tianhao Ba, Jing Wu, Xueyao Han, Linong Ji

**Affiliations:** Department of Endocrinology and Metabolism, Peking University People’s Hospital, Beijing, China

**Keywords:** diabetes, glycated albumin, HbA1c, hemolytic anemia, hereditary spherocytosis, *SLC4A1* gene

## Abstract

**Background:**

Hemoglobin A1c (HbA1c) is a critical biomarker used for the diagnosis and management of diabetes. However, nonglycemic genetic variations may affect the accuracy of HbA1c measurements.

**Methods:**

We presented a clinical evaluation of a type 2 diabetic patient with an *SLC4A1* (solute carrier family 4 member 1) gene mutation, characterized by high blood glucose and low HbA1c, and estimated the carrier frequency of *SLC4A1* variants in Chinese population.

**Results:**

A 56-year-old patient with type 2 diabetes presented with a low HbA1c level, an elevated glycated albumin percentage (GA), normal hemoglobin and albumin levels, hemolysis, and increased red blood cell osmotic fragility. Exome sequencing revealed a heterozygous mutation in *SLC4A1* gene (c.1239_1241del), which is associated with hereditary spherocytosis. Further research indicates that around 0.756% of individuals in China carry pathogenic or likely pathogenic *SLC4A1* variants.

**Conclusions:**

We report the *SLC4A1* c.1239_1241del variant, which perturbs HbA1c via nonglycemic mechanisms, likely through a reduction in the erythrocyte lifespan, and similar variants may not be rare in Chinese population.

## Introduction

Hemoglobin A1c (HbA1c), formed by a non-enzymatic glycation reaction between hemoglobin and glucose in red blood cells (1), serves as a widely utilized biomarker to estimate ambient glycemia over the preceding 2–3 months (2). It has been recognized as a key diagnostic test for diabetes and as a measure of glycemic control, as well as a clinical tool for managing the risks of complications (3). However, HbA1c, which is influenced by genetic variation through nonglycemic pathways, may not accurately reflect ambient glycemia or the risk of type 2 diabetes mellitus (T2DM) and could impact the validity of HbA1c as both a diagnostic test and a measure of glycemic control in certain individuals or populations (4–7). *SLC4A1* (solute carrier family 4 member 1) encodes erythrocyte membrane protein 3, which is critical for red blood cell membrane stability. Jin-Fang Chai et al. identified that the *SLC4A1* rs769664228 variant reduced HbA1c by 0.38 ± 0.06% in Malay individuals, likely by reducing erythrocyte lifespan (4). However, the effect of the *SLC4A1* gene on hemoglobin A1c remains incompletely understood, particularly in Chinese population, where relevant studies and case reports are lacking. We present a case demonstrating the non-glycemic effect of *SLC4A1* variants on glycated hemoglobin in Chinese individuals. Additionally, we evaluate the carrier frequency of *SLC4A1* pathogenic variants within Chinese population.

## Case description

Ethical approval was granted by Ethics Committee of Peking University People’s Hospital, and informed consent was obtained from the patient.

### Patients

Male, 56 years old, diagnosed with type 2 diabetes in 2023. His glucose-lowering regimen was initiated with a combination of metformin and glimepiride. His fasting plasma glucose (FPG) was recorded at 7.8 mmol/L, prompting a consultation at our hospital to optimize his glucose-lowering therapy in April 2024. He was diagnosed with hyperbilirubinemia and splenomegaly during a physical examination performed 20 years ago. Since then, he has undergone annual check-ups, with no significant changes observed.

In this consultation, several tests were conducted (**Table 1**). The patient’s fasting insulin levels was within the normal range, and anti-glutamic acid decarboxylase antibody testing returned negative, confirming a diagnosis of type 2 diabetes. However, the HbA1c level measured using enzymatic arrays was 4.5%, while fasting plasma glucose was 6.29 mmol/L and glycated albumin (GA) was 16.94% (normal range: 11.00-16.00%). The patient presents with a low HbA1c level, an elevated glycated albumin (GA), and normal hemoglobin and albumin levels.

**Table 1:** Laboratory results of this patient.

Test item	Laboratory findings	Reference range
GLU (0min) (mmol/L)	6.29↑	3.30-6.10
HbA1c (%)	4.5	4.00-6.00
GA (%)	16.94↑	11.00-16.00
RBC (×10^12^/L)	4.29↓	4.30-5.80
Hgb (g/L)	143	130.00-175.00
Reticulocyte (×10^6^/L)	0.3436↑	0.024-0.084
Reticulocyte (%)	8.01↑	0.50-1.50
MCV (fl)	89.7	82.00-100.00
MCH (pg)	33.3	27.00-34.00
MCHC (g/L)	371↑	316.00-354.00
RDW (%)	15.9↑	0.00-15.00
Hematocrit (%)	38.5↓	40.00-50.00
Platelet (×10^9^/L)	127	125.00-350.00
WBC (×10^9^/L)	5.68	3.50-9.50
Ferritin (ng/ml)	357	30.00-400.00
Vitamin B12 (pg/ml)	576	197.00-771.00
Folic Acid (ng/ml)	7.34	4.20-19.80
ALT (U/L)	16	9.00-50.00
AST (U/L)	18	15.00-40.00
LDH (U/L)	237	109.00-245.00
TBIL (µmol/L)	110.5↑	3.00-21.00
DBIL (µmol/L)	11.9↑	0.00-7.00
ALB (g/L)	50.1	40.00-55.00
TG (mmol/L)	0.61	0.45-1.70
LDL-C (mmol/L)	1.18↓	1.90-4.10
eGFR (ml/min×1.73m^2^)	99.13	
K (mmol/L)	4.1	3.50-5.30
INS (0min) (µIU/ml)	4.63	2.60-24.90
GAD-Ab (U/ml)	<0.50	<5
UACR (mg/g)	5.87	0.00-26.00

ALB, albumin; ALT, alanine transaminase; AST, aspartate aminotransferase; DBIL, direct bilirubin; eGFR, estimated glomerular filtration rate; GA, glycated albumin; GAD-Ab, anti-glutamic acid decarboxylase antibody; GLU, glucose; HbA1c, glycated hemoglobin; Hgb, hemoglobin; INS, insulin; K, serum potassium; LDH, lactate dehydrogenase; LDL-C, low-density lipoprotein cholesterol; MCH, mean corpuscular hemoglobin; MCHC, mean corpuscular hemoglobin concentration; MCV, mean corpuscular volume; RBC, red blood cell; RDW, red cell distribution width; TBIL, total bilirubin; TG, triglyceride; UACR, urinary albumin/creatinine ratio; WBC, white blood cell.

### Diagnostic evaluation

The elevated reticulocyte count and significantly increased direct bilirubin, along with the history of splenomegaly, indicate the presence of extravascular hemolysis in the patient. Screening for common causes of hemolytic disease, including tests for alkali-resistant hemoglobin, direct antiglobulin (Coombs) test, hemoglobin electrophoresis, isopropanol, Heinz bodies, hemoglobin H inclusion bodies, methemoglobin reduction, and the glucose-6-phosphate dehydrogenase fluorescent spot test, all yielded normal results. However, erythrocyte osmotic fragility was found to be increased. Based on these findings, autoimmune hemolysis, glucose-6-phosphate dehydrogenase deficiency, paroxysmal hemoglobinuria, and abnormal hemoglobinopathies were excluded, leading to the suspicion of hereditary spherocytosis (HS).

### Genetic analysis

**Figure 1:**
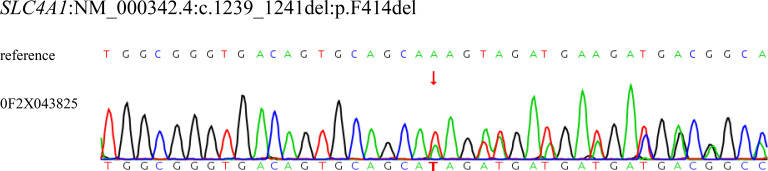
The whole exome sequencing and Sanger sequencing verification showed the patient had the heterozygous variant of *SLC4A1*; c.1239_1241del; p.F414del. *SLC4A1*, solute carrier family 4 member 1.

Whole exome sequencing (WES) confirmed the presence of a heterozygous variant in *SLC4A1* gene (c.1239_1241del, p.F414del), which was further validated by Sanger sequencing (**Figure 1**). The deletion mutation occurs in the bicarbonate transporter-like transmembrane domain (PM1), and results in a change in protein length, without affecting the open reading frame (PM4). The variant is not present in the reference populations, including the 1000 Genomes Project, the Exome Aggregation Consortium database, and the Genome Aggregation Database (PM2_Supporting). The patient’s phenotype is consistent with hereditary spherocytosis (PP4). Therefore, it could be classified as Likely-pathogenic (LP) according to the ACMG Standards and Guidelines (PM1+PM4+PM2_Supporting+PP4) (8).

### The estimation of carrier frequency of *SLC4A1* variants in Chinese population

In order to estimate the carrier frequency of *SLC4A1* variants in Chinese population, we searched the China Metabolic Analytics Project (ChinaMAP) database (www.mbiobank.com), which comprises whole exome sequencing (WES) data from 10,588 Chinese individuals, randomly selected from eight major ethnic groups across 27 provinces of China, among ~450 000 participants (9). In the ChinaMAP database, we identified *SLC4A1* variants with a minor allele frequency (MAF) < 0.005, and annotated these mutations using ANNOVAR (version release 2025-03-02) and SnpEff (v5.1). The pathogenicity of the variants was evaluated according to the American College of Medical Genetics (ACMG) recommendations (8).

A total of 1,232 *SLC4A1* variants were identified in the ChinaMAP database (**Supplementary Table 1**). There were 99 rare mutations with a MAF < 0.005, excluding synonymous mutations and mutations in noncoding regions (**Supplementary Table 2**). According to the ACMG criteria, c.2102G>A(p.G701D, allele frequency: 0.104%) and c.388G>A(p.G130R, allele frequency: 0.156%) were classified as Pathogenic. Two variants, c.1331C>A (p.T444N, allele frequency: 0.113%) and c.-68-1G>T (allele frequency: 0.005%) were categorized as LP. The other 95 variants were classified as Uncertain Significance. Mutations in *SLC4A1* gene typically cause hemolytic diseases in a dominant inheritance pattern (10, 11), but p.T444N and p.G130R have only been documented to cause red blood cell abnormalities in compound heterozygous or homozygous states. Therefore, approximately 0.756% of individuals in China carry pathogenic or likely pathogenic *SLC4A1* variants.

## Discussion

Here, we report a type 2 diabetes patient with the *SLC4A1* c.1239_1241del variant, resulting in an abnormal reduction of glycated hemoglobin via nonglycemic mechanisms. By querying the ChinaMAP database, we found that the carrier frequency of pathogenic or likely pathogenic *SLC4A1* variants is not low, indicating the need to focus on the nonglycemic impact of *SLC4A1* variants on HbA1c measurements.

Glycated hemoglobin serves as a key biomarker for indicating the mean blood glucose level over the average lifespan of an erythrocyte (~3 months in humans). GA reflects the patient’s average blood glucose level over the preceding 2–3 weeks. Established correlations confirm: FPG (mg/dl) = 21.9 × HbA1c (%) - 9.2 (r = 0.854, P < 0.01) (12). GA (%) = 2.452 × HbA1c (%) + 3.636 (r = 0.647, P < 0.01) (13). Therefore, it can be concluded that, in this case, the patient’s HbA1c level does not align with the FPG and GA measurements.

HbA1c is influenced by a range of factors, including age, ethnicity, geography, pregnancy status and so on. The lifespan of red blood cells and the type of hemoglobin are critical determinants of glycated hemoglobin levels. In cases of hemolysis and blood loss, the shortened lifespan of red blood cells leads to a reduced glycation process, resulting in abnormally low glycated hemoglobin levels. In iron-deficiency anemia, aplastic anemia, and following splenectomy, the prolonged lifespan of red blood cells extends the glycation process, leading to abnormally high glycated hemoglobin levels. Additionally, abnormalities in hemoglobin structure can interfere with the accurate measurement of glycated hemoglobin (14).

In this case, hemoglobin was measured using enzymatic arrays at our hospital, a method known for its high accuracy and reduced susceptibility to interference from abnormal hemoglobin (15). Other conditions that may lead to a low HbA1c level include hemolytic diseases, such as erythrocyte membranopathies, immunohemolytic anemia, hemoglobinopathies, erythrocyte enzymopathies, rapidly progressing type 1 diabetes, severe jaundice, hyperlipidemia, blood loss, and excessive intake of salicylates, as well as vitamins C and E, among others (16). The patient denied any history of anemia, or high-dose vitamin consumption. Upon reviewing the patient’s history of splenomegaly alongside laboratory findings of elevated reticulocyte (8.01%) and indirect bilirubin levels, we initially attributed the reduced HbA1c level to a possible hemolytic disease. Following a preliminary assessment, the patient ruled out common hereditary causes of hemolysis, with the exception of HS. Genetic sequencing was subsequently performed, revealing a heterozygous in-frame deletion in *SLC4A1* gene. Mutations in this gene have been linked to HS.

The clinical manifestations of hereditary spherocytosis exhibit considerable variability, ranging from subclinical to severe forms, which likely leads to an underestimation of the disease’s prevalence. Hereditary spherocytosis represents a group of heterogeneous disorders resulting from various molecular defects, with the common feature of weakened vertical linkages between the membrane skeleton and the lipid bilayer and its integral proteins, resulting in a reduced membrane surface area, a lower surface area-to-volume ratio, and the formation of spherocytes that have reduced deformability and are selectively retained and damaged in the spleen (10). Band 3 protein plays a crucial role in vertical connections, and mutations in *SLC4A1* gene can lead to band 3 protein deficiency, typically presenting as a mild to moderate, dominantly inherited disorder (10).

A Genome-Wide Association Study (GWAS) conducted on the Malay population in Singapore suggests that the ethnicity-specific variant *SLC4A1*:p.Ala400_Ala408del may reduce HbA1c by 0.38 ± 0.06% via erythrocyte pathways, though red blood cell-related parameters were not provided (4). The novel mutation identified in our study is located near rs769664228, within the first transmembrane domain, and is also an in-frame deletion mutation, providing some basis for the speculation made at that time. We also approximately estimated the carrier frequency of pathogenic or likely pathogenic *SLC4A1* variants in Chinese population. Future studies are warranted to investigate the erythrocyte characteristics of *SLC4A1* variant carriers and their associations with HbA1c measurement bias in Chinese biobanks. In addition to *SLC4A1* gene, several large-scale GWAS studies have identified multiple variants that influence HbA1cglycated hemoglobin via nonglycemic pathways. A 2017 GWAS study involving 159,940 individuals of European, African, East Asian, and South Asian descent identified 22 erythrocyte-related variants that were significantly associated with HbA1c at the genome-wide level. In African Americans, the X-linked *G6PD* G202A variant may lead to the omission of approximately 2% (N = 0.65 million, 95% CI 0.55-0.74) of adults with T2DM when using HbA1c screening (17). In East Asian population, the *G6PD*-Canton and *G6PD*-Kaiping variants were found to be associated with nonglycemic reductions in HbA1c (18). G6PD deficient men have a 4-year delay in type 2 diabetes diagnosis and 1.4 higher odds of microvascular complications than non-carriers (19). An Amerindian ancestry-specific variant, *HBM*-rs145546625, was associated with HbA1c and hematologic traits but not with fasting glucose (20). A common South Asian missense *PIEZO1* variant (rs76722092, MAF 3.9%) lowers HbA1c through nonglycemic effects, resulting in delayed type 2 diabetes diagnosis (21). Our case report of a novel *SLC4A1* mutation extends the spectrum of nonglycemic determinants of HbA1c beyond the common loci, and indicates the impact of *SLC4A1* variations on the diagnosis and treatment of diabetes in the Chinese population.

In conclusion, despite being internationally recommended for the diagnosis and management of T2DM, glycated hemoglobin is influenced by nonglycemic variants. Routine blood tests are recommended to identify patients for whom HbA1c is unsuitable as a long-term marker, in which case simultaneous measurement of GA and plasma glucose is advised.

## Data Availability

The data presented in the study are deposited in the Figshare repository, accession number 10.6084/m9.figshare.31901455.
